# Assessment of Diabetic Cardiomyopathy by Cardiovascular Magnetic Resonance T1 Mapping: Correlation with Left-Ventricular Diastolic Dysfunction and Diabetic Duration

**DOI:** 10.1155/2017/9584278

**Published:** 2017-07-16

**Authors:** Yongning Shang, Xiaochun Zhang, Weilling Leng, Liu Chen, Xiaotian Lei, Tianjing Zhang, Andreas Greiser, Ziwen Liang, Jian Wang

**Affiliations:** ^1^Department of Radiology, Southwest Hospital, Third Military Medical University, Chongqing, China; ^2^Department of Endocrinology, Southwest Hospital, Third Military Medical University, Chongqing, China; ^3^Northeast Asia MR Collaboration, Siemens Healthcare, Beijing, China; ^4^Siemens Healthcare, Erlangen, Germany

## Abstract

**Purpose:**

To quantify extracellular matrix expansion with the cardiovascular magnetic resonance (CMR) T1 mapping technique and the derived extracellular volume fraction (ECV) in diabetic cardiomyopathy (DbCM) patients and to detect the relationship among ECV, duration of diabetes, and diastolic function.

**Materials:**

Thirty-eight patients with diabetic cardiomyopathy (20 males, age 54.6 ± 8.6 years) and thirty-two matched normal controls (15 males, age 51.4 ± 13.6 years) were prospectively enrolled. All of them were scanned by T1 mapping to obtain the native and postcontrast T1 values of myocardium and blood, and ECV was calculated accordingly. All patients also underwent transthoracic echocardiographic tissue Doppler imaging to assess left-ventricular diastolic function.

**Results:**

There was a significant difference in ECV between the two groups (DbCMs 30.4 ± 2.9% versus controls 27.1 ± 2.4%, *P* < 0.001). The duration of diabetes was positively and strongly associated with ECV (*R* = 0.539, *P* = 0.0005). There was also a significant difference in ECV (*P* ≤ 0.001) among four groups (A, controls; B, DbCM patients with duration of diabetes <5 years; C, 5–10 years; and D, >10 years). ECV was negatively associated with LV E'/A' (*R* = −0.403, *P* = 0.012).

**Conclusion:**

CMR T1 mapping can reflect myocardial extracellular matrix expansion in DbCM and can be a powerful technique for the early diagnosis of DbCM.

## 1. Introduction

Type 2 diabetes mellitus (T2DM), one of the most common chronic diseases globally, affects nearly four hundred million individuals [1]. Diabetic cardiomyopathy (DbCM), which is present in almost two-thirds of patients with T2DM, is defined as myocardial dysfunction occurring independently of coronary artery disease, valvular dysfunction, or hypertension [2, 3].

The minimal criteria for the diagnosis of DbCM include the presence of left-ventricular (LV) diastolic dysfunction but preserved systolic function, which makes early diagnosis of DbCM difficult. Diffuse expression, accumulation, and crosslinking of interstitial collagen can expand the extracellular space of the myocardium, which contributes to the development and progression of DbCM [4]. Pathologic biopsy is a gold standard for assessing myocardial fibrosis. Whereas many techniques have been developed to assess myocardial fibrosis (such as CT, echocardiography, SPECT, and PET), these are less accurate than contrast-enhanced cardiovascular magnetic resonance (CMR) [5]. The conventional CMR late gadolinium enhancement (LGE) method, the validation, and prognostic advantages of which have been examined extensively in a variety of pathologic studies have been described as a superior tool to other imaging modalities in the identification of cardiac scars or local fibrosis [5]. Despite adequate contrast between “normal” myocardium and scar tissue, diffuse and interstitial fibrosis in many disease processes cannot be effectively detected by LGE. However, the elevated sensitivity of T1 mapping has proven useful in these cases [5, 6].

Novel cardiac MR T1 mapping techniques make it feasible to measure myocardial extracellular matrix (ECM) expansion in vivo. Extracellular volume fraction (ECV), derived from native-T1 mapping and postcontrast T1 mapping after the administration of a bolus of gadolinium (Gd) contrast, can quantify myocardial ECM expansion. ECV quantification correlates well with histology [7] and is highly reproducible [8] and sensitive [9]. For those reasons, ECV has been regarded as an essential biomarker for quantifying the full spectrum of diffuse myocardial fibrosis. In the DbCM setting, the relationships among mapping parameters (including native T1 value, postcontrast T1 value, and ECV) and the duration of diabetes and diastolic function should be elucidated.

The specific purpose of the present study was to quantify ECM expansion with the cardiac MR T1 mapping technique and the derived ECV in DbCM, detect the relationship between ECV and the duration of diabetes, and evaluate the relationship between ECV and diastolic function.

## 2. Materials and Methods

### 2.1. Study Patients

This study was approved by the Institutional Review Board (IRB) of our hospital, and all subjects gave informed consent. This study was conducted in our hospital between June 2015 and September 2016. The inclusion criteria for DbCM patients included (1) an initial diagnosis of T2DM according to the criteria of the World Health Organization [10]; (2) left-ventricular early diastolic dysfunction (echocardiographic Doppler Tissue Imaging E'/A' < 1, derived from the ratio of early to late diastolic mitral annulus velocity [11]); (3) no history of symptomatic coronary heart disease (no clinical manifestation and no abnormal signal on first pass perfusion and late gadolinium enhancement images); and (4) no hypertension. Exclusion criteria included glomerular filtration rate (GFR) ≤30 mL/min/1.7 m^2^ and other standard contraindications to cardiac MR. Forty consecutive participants were recruited for cardiac MR examination. Two participants were excluded because of the presence of high heart rate (>100 beats/min) during physical examination. Thirty-two normal controls were recruited from the community population during the same period.

### 2.2. Anthropometry and Biochemistry Exams

All DbCM patients and normal controls underwent height, weight, and blood pressure examinations. Blood samples were collected at about 30 minutes before scanning. Those blood samples were immediately sent to the Department of Clinical Laboratory and Nuclear Medicine and immediately analyzed to obtain haematocrit (HCT), serum glucose, glycated hemoglobin (HbA1c), insulin, C-peptide, creatinine, cystatin C, triglycerides (TG), total cholesterol (TC), high-density lipoprotein cholesterol (HDL) and low-density lipoprotein cholesterol (LDL) levels, myocardium zymogram examination, and markers of myocardial damage.

### 2.3. Echocardiography

All patients underwent transthoracic echocardiography at rest using a commercially available ultrasound machine and transducer (Philips IE33 color Doppler scanner, S5-1 heart probe, the Netherlands). According to the guidelines of the American Society of Echocardiography [11], tissue Doppler imaging (TDI) was used to measure left-ventricular diastolic function. The sample volume was placed at the mitral valve annulus in an apical four-chamber view. The septal mitral valve annulus early E' wave (passive left-ventricular filling) and late A' wave (atrial contraction) velocity and the ratio of the two (E'/A') were assessed.

### 2.4. Cardiac MR Protocol

All DbCM patients and normal controls underwent cardiac MR examination on a 3T system (MAGNETOM Trio Tim, Siemens Healthcare, Erlangen, Germany). They were placed in a supine position using a 6-channel body matrix plus 2 rows of the spine array. Breath holding after expiration and electrocardiographic gating were used when necessary. LGE was performed about 15 min after bolus injection of 0.2 mmol/kg i.v. gadoteric acid meglumine bolus (Dotarem, Guerbet, BP7400, F95943, Roissy CdG Cedex, France) with the Phase Sensitive Inversion Recovery sequence (TR/TE 680/1.94 ms, slice thickness 8 mm, spacing between slices 1.6 mm) to exclude local myocardial fibrosis.

### 2.5. T1 Mapping

T1 mapping was performed using a modified Look-Locker Inversion Recovery (MOLLI) prototype sequence (Siemens Work-in-Progress no. 448B, (VB17A)) [12] (FOV 400 × 300 mm^2^, matrix 256 × 166, 6 mm thickness, TR/TE 301.7/1.09 ms, flip angle 35 degrees, 6/8 partial-Fourier *k*-space sampling, PAT factor 2). Spacing in between inversion experiments and image acquisition were both heartbeat-based (precontrast T1 recovery: a 5 (4) 2 sampling scheme, trigger delay 80 msec; postcontrast T1 recovery: a 4 (1) 2 (2) 2 sampling scheme, trigger delay 160 msec). Native T1 mapping was performed at basal, middle, and apical short axis of left-ventricular slices. Postcontrast T1 mapping was performed 20 min after bolus injection at the identical slice positions. T1 maps were generated online immediately after the scan. The motion correction (MoCo) technique was used.

### 2.6. Cardiac MR Analysis

#### 2.6.1. T1 Mapping

**Figure 1:**
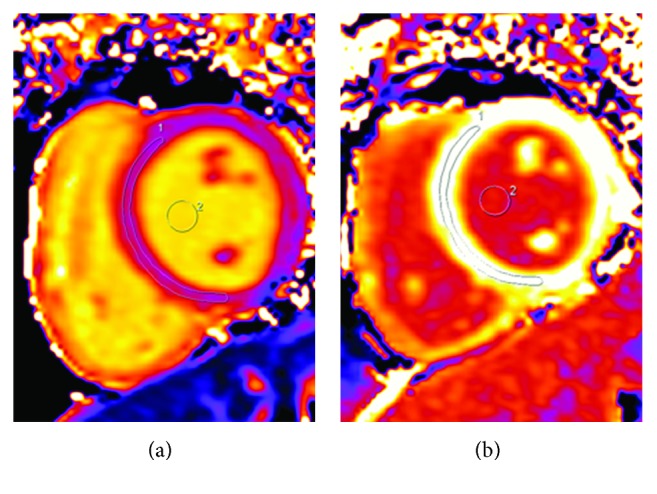
ROIs on native (a) and postcontrast (b) T1 mapping.

T1 mapping images were analyzed by two blinded radiologists (observer 1, 12 years of experience; observer 2, 10 years of experience) with a multimodality processing platform (syngo MR, Siemens Healthcare, Erlangen, Germany). The region of interest (ROI) was selected manually on the middle third of interventricular septum myocardium to avoid epicardial and endocardial partial-volume effects. The other ROI in the left-ventricular blood pool was located far away from both papillary muscles and myocardium to avoid partial-volume effects (Figure 1). The ECV of each slice was calculated with the following formula [13]:

(1) $$\begin{array}{c} ECV=\left(1-haematocrit\right)\frac{\left(1/T1\;myo\;post\right)-\left(1/T1\;myo\;native\right)}{\left(1/T1\;blood\;post\right)-\left(1/T1\;blood\;native\right)}, \end{array}$$

where “myo” means myocardium. We averaged ECV measures of basal, middle, and apical short-axis images to obtain the final ECV value.

### 2.7. Statistical Analysis

Categorical variables were presented as percentages. Continuous variables were summarized as the mean and standard deviation (SD). Independent *t*-tests were used to evaluate the differences between the DbCM group and the normal control group; the Mann–Whitney *U* test was performed when standard variance was heterogeneous. One-way ANOVA was performed to test the differences among the four groups, and the Bonferroni test was used as a post hoc test. The Kolmogorov-Smirnov test was used as a normality test. The relationships between bivariate were analyzed using Pearson's or Spearman's method. Statistical tests were two-tailed, and *P* < 0.05 was considered statistically significant. Statistical analysis was performed using commercially available software (SPSS for Windows 21.0, Inc, Chicago, IL, USA).

## 3. Results

The results of anthropometry and biochemistry assessments are shown in Table 1.

Thirty-eight patients (20 males, age 54.6 ± 8.6 years, the duration of diabetes 7.0 [2.8–11.0] years) with DbCM and thirty-two normal controls (15 males, age 51.4 ± 13.6 years) were studied. Fasting blood glucose and HbA1c were higher in patients with DbCM than in normal controls. DbCM was also associated with higher TG, HDL, aspartate aminotransferase (AST), ischemia modified albumin (IMA), and troponin levels. There were no significant differences between the two groups in age, gender, height, weight, and so on (all *P* > 0.05).

The results about standard cardiac MR parameters on morphology (LV end diastolic volume, end systolic volume, and mass) and systolic function have been published in our previous paper [14]. There were no visually observed abnormal signal on LGE images of all participants, suggesting no local myocardial scar.

**Figure 2:**
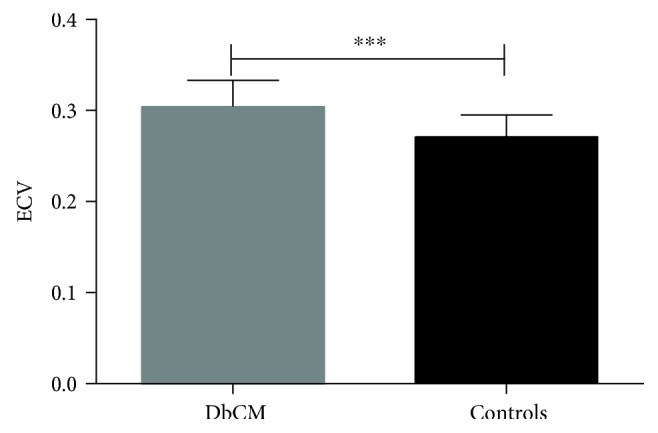
Differences in left-ventricular ECV between patients with DbCM and control subjects. ∗∗∗ means *p* < 0.001.

DbCM patients and controls had similar myocardial native T1 values (DbCMs 1213.5 ± 57.5 ms versus controls 1212.8 ± 41.4 ms, *P* = 0.950) and postcontrast T1 values (DbCMs 518.8 ± 45.1 ms versus controls 528.9 ± 38.9 ms, *P* = 0.324). However, there was a significant difference in ECV between the two groups (DbCMs 30.4 ± 2.9% versus controls 27.1 ± 2.4%, *P* < 0.001, Figure 2), indicating the presence of extracellular volume expansion in the myocardium of patients with DbCM.

Table 2 shows Pearson's correlation and Spearman's correlation analysis of ECV to biochemical characteristics, which are typically different in DbCM. ECV correlated significantly with insulin.

**Figure 3:**
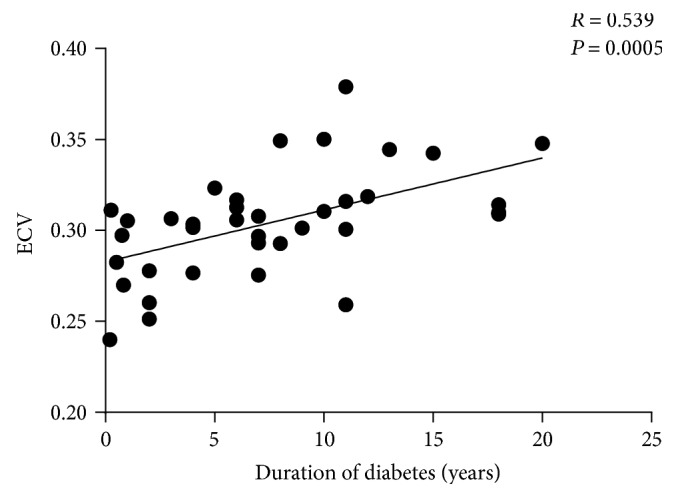
Relationship between ECV and the duration of diabetes.

**Figure 4:**
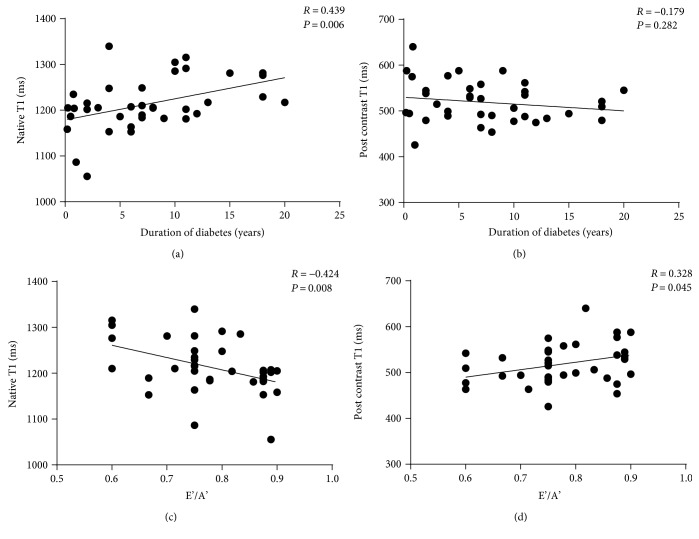
Correlations among myocardial T1 value, left-ventricular TDI E'/A', and duration of diabetes. (a) Correlation between native myocardial T1 value and duration of diabetes. (b) Correlation between postcontrast myocardial T1 value and duration of diabetes. (c) Correlation between native myocardial T1 value and TDI E'/A'. (d) Correlation between postcontrast myocardial T1 value and TDI E'/A'.

Pearson's correlation analysis showed that ECV was positively associated with the duration of diabetes (*R* = 0.539, *P* = 0.0005; Figure 3), and multivariable stepwise linear regression indicated that the duration of diabetes was an independent and strong predictor of ECV (Table 3). Native T1 value was positively associated with the duration of diabetes in univariable correlation analysis (*R* = 0.439, *P* = 0.006, Figure 4(a)) but not in multivariable correlation analysis when adjusted for insulin, age, height, weight, BMI, BSA, systolic blood pressure, and diastolic blood pressure (*R* = 0.157, *P* = 0.548). Postcontrast T1 value did not correlate with the duration of diabetes in uni- and multivariable correlation analyses (*R* = −0.179, *P* = 0.282; *R* = 0.211, *P* = 0.415, respectively, Figure 4(b)).

Based on the duration of diabetes (7.0 [2.8–11.0] years), all DbCM patients were divided into three groups (group B, <5 years; group C, 5–10 years; and group D, >10 years). There was a significant difference in ECV (ANOVA *P* ≤ 0.001, Table 4) among controls (group A) and groups B, C, and D. Nevertheless, native and postcontrast myocardial T1 values were not significantly different among four groups (*P* = 0.086 and *P* = 0.596, resp., Table 4).

**Figure 5:**
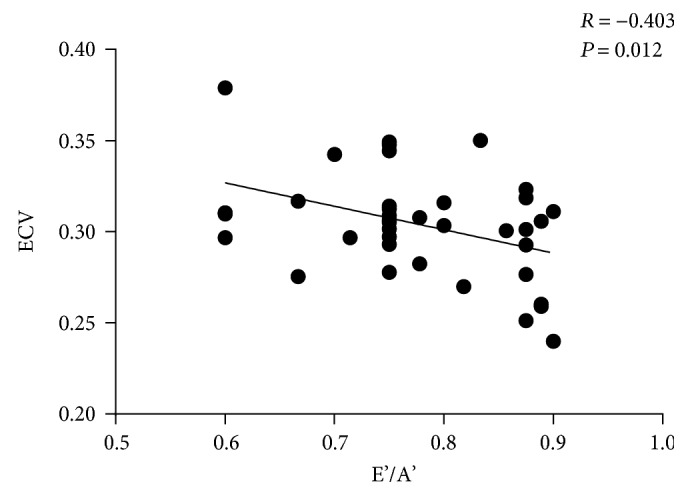
Relationship between ECV and left-ventricular TDI E'/A'.

All patients with DbCM exhibited decreased echocardiographic TDI mitral annulus velocity E'/A' (all E'/A' < 1), and Pearson's correlation analysis showed that ECV was negatively associated with E'/A' (*R* = −0.403, *P* = 0.012; Figure 5). E'/A' also correlated with native T1 value (*R* = −0.424, *P* = 0.008, Figure 4(c)) and postcontrast T1 (*R* = 0.328, *P* = 0.045, Figure 4(d)). We previously showed that there was no significant difference in EF between DbCM patients and controls [14]. Here, we found that EF did not correlate with ECV in DbCM patients (*R* = −0.023, *P* = 0.892).

## 4. Discussion

DbCM is defined as myocardial dysfunction that occurs independently of coronary artery disease, valvular dysfunction, or hypertension [2, 3]. DbCM is associated with altered left-ventricular geometry, function, and tissue characterization [13, 15, 16]. Using cardiac MR, we found that (1) DbCM is accompanied by increased ECV, which indicates extracellular volume expansion; (2) ECV is associated with the duration of diabetes; and (3) increased ECV correlates with reduced TDI E'/A'.

Although native T1 and postcontrast T1 did not statistically differ between DMs and controls, there was a trend that native T1 was longer and postcontrast T1 was shorter in DMs. As we know, ECV was calculated as (1 − HCT)∗(1/(T1 myo post) − 1/(T1 myo native))/(1/(T1 blood post) − 1/(T1 blood native)). Combination of the differences of native T1 and post contrast T1 may lead to statistical difference in ECV. It demonstrated that ECV may be more sensitive than native and postcontrast T1 to indicate DM-related myocardial change. This might have significant clinical implications for diseases that are characterized by an alteration in ECV, such as DbCM. With increased sensitivity, the disease could potentially be detected earlier when only subtle differences in ECV may be present.

Several studies have shown that DbCM is associated with increased collagen, especially type III collagen, in the myocardial interstitium of humans [17] and animal models [18]. However, previous studies have reported controversial results about the relationship between DbCM and ECM expansion [19, 20]. ECV, derived from T1 mapping cardiac MR, is considered as the biomarker of cardiac fibrosis, which is caused by the deposition of perivascular and intermyofibrillar collagen [21]. DbCM is expected to be associated with increased ECV. However, previous studies presented conflicting results on the elevation of ECV in patients with T2DM or DbCM. Khan et al. [16] and Levelt et al. [15] reported that patients and control subjects had similar ECVs, but the patient population in the former study consisted of young adults, and the patient population of the latter study was too highly selected (no diabetic complications or Hba1C >9%). Wong et al. [13] indicated that diabetes (accompanied by 85% hypertension, 33% prior coronary revascularization, and 11% prior acute myocardial infarction) was associated with higher ECVs than nondiabetes. Other factors, such as hypertension, may influence the study results. Therefore, in the current study, we carefully selected patients and normal control subjects. We found that DbCM was associated with elevated ECV, despite the fact that patients with DbCM had similar native and postcontrast T1 values as healthy control subjects, which was consistent with a previous study [16]. This may indicate the presence of diffuse myocardial fibrosis in patients with DbCM.

Interestingly, we found that duration of diabetes, not hyperglycemia, HbA1c, troponin, or IMA, was an independent predictor of ECV and group with the longer duration of diabetes was associated with higher ECV value. Recent studies showed that hyperglycemia, even transient hyperglycemia (a few hours), can cause persistent (a few days) damage to cells by disrupting the signal feedback loop [22] and hyperglycemia can sufficiently promote the proliferation of cardiac fibroblast [23]. HbA1c is a marker of time-averaged glucose level. Neither glucose nor HbA1c can reveal the severity of long-term damage to the myocardium. Elevated troponin and IMA may indicate an impairment of cardiac myocytes, but these markers cannot reflect the severity of long-term damage. The relationship between diabetic history and ECV indicates that ECV may represent the long-term effects of DbCM on the myocardium. This should be confirmed by further studies with more participants.

Our results show that there is a significant relationship between impaired LV diastolic function and increased ECV in patients with DbCM, although the association between ECV and diastolic dysfunction has been reported in patients with heart failure with preserved ejection fraction [24] and in children and young adults with congenital aortic stenosis [25]. The findings concerning the relationship between LV function and fibrosis are in agreement with those of previous studies. On the one hand, elevated collagen content in the myocardium affects cardiac relaxation. Using a rat model, Mizushige et al. [26] showed that diabetes was associated with low transmitral inflow velocity of early filling rate, high late filling rate, and the severity of the impairment becoming worse with disease progression. Elevated myocardial collagen content is closely associated with impaired left-ventricular diastolic function [23]. On the other hand, the formation of advanced glycosylation end products on the myocardium induces collagen molecules in situ to cross-link with each other, which leads to the impaired elasticity of collagen and a subsequent decrease in myocardial compliance [27]. Increased myocardial stiffness along with diabetes progression induces impaired LV diastolic function.

There are some limitations in this preliminary study. First, the overall number of subjects was limited; thus, the inhomogeneity of the patient group may have introduced bias. Second, we did not recruit patients with a GFR ≤30 mL/min/1.7 m^2^, and the patients in the study received different treatments. Finally, a myocardium zymogram examination was performed, markers of myocardial damage were assessed in only 84% of the patients and controls, and urine microalbumin was examined in only 74% of individuals.

## 5. Conclusion

When compared to normal control participants matched to age, BMI, and BSA, patients with DbCM exhibited elevated ECV detected using cardiovascular magnetic resonance T1 mapping technique. LV ECV correlates well with the duration of diabetes and LV impaired diastolic function. Cardiovascular magnetic resonance T1 mapping can reflect myocardial extracellular matrix expansion in patients with diabetic cardiomyopathy and may be a powerful technique for early diagnosis of diabetic cardiomyopathy.

## Figures and Tables

**Table 1:** Anthropometry and biochemical characteristics.

	DbCM (*N* = 38)	Control (*N* = 32)	*P* value
*Anthropometry*			
Age, y	54.6 ± 8.6	51.4 ± 13.6	0.229
Diabetic history, y	7.0 [2.8–11.0]		
Male, *n* (%)	20 (52.6)	15 (46.9)	0.405
Height, m	1.62 ± 0.07	1.61 ± 0.07	0.711
Weight, kg	63.9 ± 9.8	61.3 ± 10.5	0.296
Body mass index, kg/m^2^	24.3 ± 2.7	23.5 ± 3.1	0.248
Body surface area, m^2^	1.68 ± 0.15	1.65 ± 0.17	0.491
Systolic blood pressure, mmHg	118.5 ± 10.5	119.0 ± 11.4	0.867
Diastolic blood pressure, mmHg	80.9 ± 5.9	83.4 ± 7.6	0.118

*Biochemical exam*			
Urine microalbumin, mg/dL	0.3 [0.1–3.0]	0.9 [0.6–1.7]	0.072
Blood urea nitrogen, mmol/L	5.8 [4.8–7.0]	5.8 [5.1–6.8]	0.810
Creatinine, *μ*mol/L	60.0 [54.6–71.6]	64.0 [57.3–76.3]	0.632
Cystatin C, mg/L	0.81 ± 0.20	0.76 ± 0.16	0.256
AST, IU/L	29.3 [23.2–35.4]	20.2 [17.3–27.6]	**0.001**
LDH, IU/L	188.1 ± 54.0	196.4 ± 46.0	0.549
*α*-HBDH, IU/L	126.0 ± 28.7	131.0 ± 37.9	0.581
CK, IU/L	93.7 [62.2–130.5]	88.5 [66.2–105.2]	0.576
Ischemia modified albumin, U/mL	82.6 ± 7.7	73.6 ± 5.7	**≤0.001**
Total cholesterol, mmol/L	5.4 [4.0–6.3]	5.2 [4.8–5.9]	0.926
Triglycerides, mmol/L	2.1 [1.1–3.6]	1.4 [0.9–2.0]	**0.013**
HDL, mmol/L	1.2 [1.0–1.3]	1.4 [1.2–1.7]	**0.001**
LDL, mmol/L	3.4 [2.5–3.9]	3.3 [3.1–3.7]	0.440
Glucose, mmol/L	8.2 [6.5–10.6]	5.3 [4.8–5.8]	**≤0.001**
Glycated hemoglobin, %	7.4 [6.7–8.9]	5.6 [5.4–5.8]	**≤0.001**
Troponin, 10^−3^ *μ*g/L	8 [5–12]	5 [3–6]	**0.001**
Myoglobin, ng/mL	26.5 [21.0–41.3]	30.4 [22.0–38.5]	0.636
Insulin, *μ*IU/mL	18.5 ± 9.8	12.8 ± 4.5	**0.021**
C peptide, ng/mL	1.8 [1.3–2.6]	1.1 [0.8–1.8]	**0.016**
Haematocrit, %	39.8 ± 4.4	41.8 ± 4.5	0.055

*Medications*			
Angiotensin-converting enzyme inhibitors, *n* (%)	5 (13.2)		
Statin, *n* (%)	26 (68.4)		
Aspirin, *n* (%)	4 (10.5)		

*Echocardiography*			
Doppler mitral annulus velocity E'/A'	0.78 ± 0.09		

AST: aspartate aminotransferase; LDH: lactate dehydrogenase; *α*-HBDH: alpha-hydroxybutyrate dehydrogenase; CK: creatine kinase; HDL: high-density lipoprotein cholesterol; LDL: low-density lipoprotein cholesterol.

**Table 2:** Relationship between ECV and biochemical characteristics.

	AST	TG	HDL	Glu	HbA1c	Troponin	C-peptide	IMA	Insulin
Rho	0.077	−0.116	−0.135	−0.138	0.025	−0.341	−0.108		
*R*								0.239	0.486
*P*	0.659	0.488	0.418	0.409	0.882	0.060	0.600	0.250	**0.014**

Rho: Spearman's correlation analyzed for nonnormal distributions. *R*: Pearson's correlation analyzed for normal distributions; AST: aspartate aminotransferase; TG: triglycerides; HDL: high-density lipoprotein cholesterol; Glu: glucose; HbA1c: glycated hemoglobin; IMA: ischemia modified albumin.

**Table 3:** Uni- and multivariable correlation analyses between ECV and the duration of diabetes.

Variable	*R*	*P* value
The duration of diabetes	0.539	**≤0.001**
The duration of diabetes, insulin	0.464	**0.022**
The duration of diabetes, insulin, age, height, weight, BMI, BSA, systolic blood pressure, and diastolic blood pressure	0.604	**0.010**

**Table 4:** Left-ventricular myocardial T1 value and extracellular volume in controls and DbCM patients.

	Controls (A, *n* = 32)	<5 years (B, *n* = 13)	5–10 years (C, *n* = 14)	>10 years (D, *n* = 11)	*P* value
Native myocardial T1 value, ms	1212.8 ± 41.4	1191.9 ± 71.0	1209.7 ± 43.1	1244.1 ± 46.1	0.086
Postcontrast myocardial T1 value, ms	528.9 ± 38.9	527.8 ± 56.7	515.5 ± 44.6	512.2 ± 30.3	0.596
Extracellular volume fraction, %	27.1 ± 2.4	28.3 ± 2.3	30.9 ± 2.1	32.2 ± 3.1	**≤0.001**
